# Energy infrastructure construction and enterprises’ Artificial Intelligence development: Evidence from China’s ultra-high voltage transmission projects

**DOI:** 10.1371/journal.pone.0355269

**Published:** 2026-10-01

**Authors:** Wenhe Lin, Yumin Guo, Bingxuan Wang, Qiuwang Cheng

**Affiliations:** College of Economics and Management, Fujian Agriculture and Forestry University, Fuzhou, China; Southwestern University of Finance and Economics, CHINA

## Abstract

Ultra-high voltage (UHV) transmission projects are a key component of China’s energy supply-side reforms, improving the efficiency and reliability of the power system. While their macro-level importance is well recognized, evidence on their impact on firm-level artificial intelligence (AI) development remains limited. This study addresses this gap by exploiting the staggered rollout of UHV transmission projects across prefecture-level cities as a quasi-natural experiment. Using a multi-period difference-in-differences (DID) approach on a panel of Chinese listed enterprises from 2007 to 2023, this study finds that UHV transmission projects significantly promote enterprises’ AI development. The effect is stronger for non-state-owned enterprises, enterprises located in inland regions, enterprises with CEO-chair duality, and high-tech enterprises. Mechanism analyses show that UHV transmission projects enhance enterprises’ AI development by strengthening technological innovation, expanding enterprises’ organizational resource base, and improving regional financial development. Further analyses indicate that UHV transmission projects also improve resource allocation efficiency and alleviate development bottlenecks. By linking physical energy infrastructure with firm-level AI development, this study highlights the importance of reliable electricity supply for technology adoption and digital transformation.

## 1. Introduction

Driven by the new wave of technological revolution, new infrastructure represented by 5G base stations, large-scale data centers, and UHV transmission projects, along with AI, has become a core focus in the field of academic research and policy making [1,2]. The expansion of such infrastructure provides essential support for new sources of regional economic growth [3]. At the same time, rising global and regional uncertainty associated with financial crises, geopolitical tensions, international trade frictions, and public health emergencies has increased the importance of infrastructure investment as a means of stabilizing economic growth and supporting technological upgrading [4].

Data centers, as the core infrastructure supporting AI operations, are experiencing rapidly growing electricity demand. As noted in the Energy and AI Report published by the International Energy Agency in April 2025, worldwide electricity consumption by data centers totalled 415 TWh in 2024, accounting for approximately 1.5% of total global electricity demand [5]. Additionally, with the rapid increase in AI training and inference workloads, this figure is projected to rise to approximately 945 TWh by 2030. Over the past decade, around 1.5 million kilometers of new transmission lines have been deployed worldwide. However, limited transmission capacity still acts as a major bottleneck restricting the expansion of power systems and undermining energy security [6]. As a key pillar of China’s new infrastructure system, UHV transmission projects can effectively alleviate the shortage of regional power supply arising from the uneven spatial distribution of energy resource endowments. UHV transmission refers to the power delivery technologies operating at voltage levels of 1000 kV and above for alternating current (AC) and 800 kV and above for direct current (DC). Compared with conventional transmission technologies, UHV transmission offers higher transmission efficiency and stronger cross-regional power transfer capability. These characteristics make it particularly suitable for China’s energy structure, where electricity generation is concentrated in the western and northern regions, while economic activities and electricity load demand are concentrated in the eastern and southern regions [7]. Relying on the UHV transmission projects, abundant renewable energy sources, including hydro, wind and solar power generated in western China, can be delivered to major electricity demand centers in central and eastern China, where supply-demand imbalances are more pronounced. This effectively mitigates structural imbalances in regional energy allocation and facilitates the optimization and transformation of the energy structure [1].

Building on this basis, we view stable and reliable power supply as a fundamental production input for enterprises’ AI development. Unlike traditional information technologies, enterprises’ AI development covers multidimensional processes, including algorithm R&D, computing power deployment, application scenario landing, and organizational capability reconstruction, rather than a single technological innovation activity [8,9]. A stable and sustainable power supply not only provides a fundamental guarantee for the operation of computing systems and intelligent production [10], but also reduces the risk associated with irreversible AI-related investment, thereby strengthening the investment incentive for the long-term deployment and AI development in the layout of enterprises. Existing studies show that UHV transmission projects improve the spatial allocation of electricity resources and the stability of cross-regional power supply [11], while greater grid flexibility can reduce fluctuations in electricity prices and supply capacity [12]. These characteristics suggest that energy infrastructure may affect enterprises’ AI development by changing the technological conditions for computing-intensive activities, influencing enterprises’ internal resource allocation, and improving the stability of the external operating environment. This multidimensional relationship provides a natural basis for organizing the subsequent mechanism analysis within the technology-organization-environment (TOE) framework. Therefore, we put forward the core research question: Can the construction and operation of UHV transmission projects translate into the observable AI development effect at the enterprise level?

A large body of literature has examined the economic and social consequences of infrastructure construction, particularly transportation and digital communication infrastructure. The existing literature has predominantly focused on transportation infrastructure and digital communication infrastructure. Studies generally find that high-speed rail, 5G deployment, and the Broadband China initiative contribute to enterprises’ technological upgrading [13,14]. In contrast, as the underlying condition to support the operation of digital technologies, the role of energy infrastructure, especially UHV transmission projects, in enterprises’ AI development has not been fully explored.

In developed economies, a stable power supply is often regarded as the established prerequisite for enterprise operations [15]. However, in emerging economies characterized by pronounced spatial mismatches between energy production and consumption, issues such as supply continuity, system stability, and cost volatility are considerably more severe than in standard production environments [16]. The lack of stability and cost constraints of power supply are exactly the core bottlenecks that restrict enterprises’ AI development. Existing studies in engineering technology and energy economics have confirmed that UHV transmission projects markedly boost the operational stability and system resilience of cross-regional power networks, and reduce the marginal power consumption cost of enterprises by enhancing the efficiency of cross-regional energy allocation [17,18]. Yet micro-level causal evidence on whether and how these improvements affect enterprises’ AI development remains limited.

Meanwhile, existing literature investigating the socioeconomic consequences of UHV transmission projects has produced mixed conclusions, with scholars documenting both positive and negative effects. At the positive effect level, the investment of UHV construction enlarges the overall scale of the power market and triggers multiplier effects on aggregate investment, contributing to employment growth and regional economic development [19]. It also improves power system efficiency and facilitates the cross-regional substitution of clean energy, thereby enhancing both enterprise productivity and environmental performance. At the potential risk level, some studies point out that the unbalanced UHV deployment may trigger the mismatch between regional resources and income, aggravate the imbalance of regional development, and even make the energy-rich but backward export regions face the risk of resource curse, hindering the diversification and upgrading of regional industries. These divergent findings further highlight the necessity of clarifying the economic benefits and mechanism of UHV transmission projects on enterprises’ AI development from the micro-enterprise level.

Against this background, we center on three key research questions: (1) Do UHV transmission projects significantly promote enterprises’ AI development? (2) If there is a promoting effect, what is the internal mechanism through which UHV transmission projects drive enterprises’ AI development? (3) Does this effect exhibit heterogeneity, and can it be reflected in improvements in energy market efficiency? Addressing these questions can help broaden the analytical horizon for exploring the nexus between energy infrastructure and enterprises’ AI development. It also clarifies the micro-level mechanisms through which large-scale energy projects influence enterprises’ intelligent transformation. More broadly, we offer empirical evidence for the collaborative formulation of energy infrastructure construction strategies and AI development policies in China as well as other developing countries.

Our study makes the following contributions: (1) Existing studies have examined enterprises’ AI development from demand-side factors such as market competition and consumption upgrading [20,21], as well as institutional and business environments [22]. However, energy infrastructure as a fundamental supply-side condition has received relatively limited attention in explaining the development of enterprise AI. Using the quasi-natural experiment created by UHV transmission projects in China, this study contributes to the literature on both energy infrastructure and AI by examining how large-scale energy infrastructure construction shapes enterprises’ AI development from a micro-level causal perspective. (2) Prior research has also emphasized firm-specific characteristics, such as managerial traits and digital capabilities [23], and policy instruments, such as intellectual property protection and R&D subsidies [24]. This study complements these perspectives by examining how external energy supply conditions promote enterprises’ AI development through reduced supply uncertainty and a more stable long-term investment environment. (3) Motivated by the multidimensional dependence of AI development on technological conditions, organizational resources, and the external environment, we employ the TOE framework to organize the mechanism analysis. We further explore the economic effects of UHV transmission projects in terms of misallocation correction and shortboard complementing, thereby expanding research on energy infrastructure and enterprises’ intelligent transformation.

The rest of this paper is structured as follows. The Literature review and research hypotheses section sorts out the relevant literature and institutional background and develops the hypotheses. The Methodology section illustrates the data sources and empirical research design. The Empirical results section presents the main results. The Further analyses section conducts further analyses. The Conclusions and implications section summarizes the conclusions and policy implications of this study.

## 2. Literature review and research hypotheses

### 2.1. Policy background

China exhibits a significant spatial imbalance between energy endowments and electricity demand. Approximately 76% of coal resources, 80% of hydropower capacity, 80% of wind power potential, and 90% of solar energy resources are concentrated in the northwest and southwest regions, whereas more than 70% of electricity consumption is located in the central and eastern areas. The primary energy production centers are roughly 1,000–4,000 kilometers away from major electricity load centers. Under the traditional provincial and local balancing system, this spatial separation constrained cross-regional electricity allocation and made it difficult to accommodate growing electricity demand.

Cross-regional power transmission consequently became an important component of China’s energy infrastructure strategy. The West-to-East Power Transmission Strategy initiated the development of a nationwide power allocation framework, but the conventional 500 kV transmission system gradually became insufficient for long-distance and large-capacity electricity transmission. Compared with conventional transmission technologies, UHV transmission provides greater transmission capacity and distance while reducing line losses and unit transmission costs. Against this background, China began to promote UHV transmission projects in 2004.

The development of UHV transmission projects in China progressed from technological demonstration to large-scale network construction. During 2005–2010, UHV technology underwent intensive testing and demonstration, culminating in the commissioning of the Jindongnan-Jingmen 1,000 kV UHV AC project in 2009 and the Xiangjiaba-Shanghai ±800 kV UHV DC project in 2010. Large-scale construction accelerated after 2011 as UHV became an important component of China’s cross-regional power grid. Further expansion after 2014 substantially strengthened interregional transmission capacity, while more recent construction has increasingly supported renewable energy integration and the development of the new-type power system. By the end of 2025, China had commissioned 46 UHV AC and DC projects, comprising 22 AC and 24 DC projects, with a total transmission line length exceeding 62,000 kilometers. Detailed project information is reported in Appendix.

An important feature of UHV development is that projects were commissioned in different regions at different points in time. Rather than being introduced nationwide simultaneously, the UHV network expanded gradually through successive batches of transmission projects. This staggered rollout generated substantial temporal and spatial variation in cities’ access to the UHV network, providing a quasi-natural experimental setting for identifying its economic effects.

By improving cross-regional electricity allocation, UHV transmission projects can increase power supply reliability and reduce the constraints associated with long-distance electricity transmission. These changes are particularly relevant to enterprises’ AI development, which relies heavily on computing infrastructure, continuous electricity consumption, and a stable power supply. Therefore, the phased expansion of the UHV network provides both an economic basis for examining its influence on enterprises’ AI development and an empirical basis for the identification strategy used in this study.

### 2.2. Literature review

Infrastructure serves as a cornerstone of economic growth by enabling spatial restructuring of economic activities and enhancing the efficiency of production factor allocation [25,26]. At the micro level, infrastructure can influence technology adoption and digital transformation by reducing transaction costs, facilitating factor flows, and improving the stability of the production environment [27]. Against the backdrop of the fast-growing digital economy, the deployment of AI technology in enterprises has gradually become a core engine for improving productivity and reshaping business models [28]. Unlike traditional information technologies, AI relies more significantly on computing resources, power supply, and operation stability, making its development highly dependent on stable and predictable infrastructure support [29].

Existing studies have mainly examined enterprises’ AI development and digital transformation from the perspectives of information infrastructure and enterprise characteristics. Broadband networks and digital infrastructure facilitate technology adoption and productivity growth by reducing information acquisition costs and improving data-processing capacity [30,31], while firm size, human capital, and organizational capabilities also shape enterprises’ ability to adopt and apply AI [32,33]. These studies show that AI development depends not only on technological infrastructure but also on enterprises’ internal capacity to organize and deploy resources. However, compared with information infrastructure, the role of energy infrastructure has received relatively limited attention, even though electricity is essential to the computing and data-processing activities underlying AI development.

Electricity constitutes a critical production input for AI-related activities. AI model training and deployment are energy-intensive and require continuous operation, making them sensitive to electricity reliability and cost conditions [34]. Power shortages and outages can distort enterprises’ production decisions and technology adoption, particularly in developing economies [35]. However, the energy infrastructure literature has focused mainly on productivity, employment, and firm performance [19,35]. In China, existing studies show that UHV transmission projects improve cross-regional energy allocation, enhance power supply stability, and contribute to regional economic development [3]. Yet firm-level evidence on whether these improvements translate into enterprises’ AI development, and through which channels, remains limited.

Taken together, the literature suggests that enterprises’ AI development is jointly shaped by the technological conditions supporting computing activities, enterprises’ organizational capacity to allocate resources, and the stability of the external operating environment. This multidimensional dependence also indicates that the effects of energy infrastructure cannot be fully understood from the perspective of electricity supply alone. Accordingly, this study uses the staggered rollout of UHV transmission projects across China as a quasi-natural experiment to examine whether and how improvements in energy infrastructure affect enterprises’ AI development. By linking macro-level energy infrastructure with firm-level AI activities, we extend existing literature on the role of energy infrastructure in the digital economy and provide micro-level causal evidence on its underlying channels.

### 2.3. Research hypotheses

As a fundamental condition supporting economic activity, energy infrastructure shapes enterprises’ technology adoption and production decisions by affecting factor availability and transaction costs [27,36]. This role becomes particularly important in the digital economy, where AI has increasingly been applied to improve production efficiency and operational decision-making [37]. Compared with traditional information technology, AI is more dependent on computing resources and power supply, and its operational cycle is defined by intensive energy demand and long-running sustainability requirements [29]. Consequently, AI development is particularly sensitive to the availability and reliability of energy infrastructure.

When electricity supply is insufficient or unstable, enterprises face greater production uncertainty and operating risks. To maintain routine production, they may prioritize electricity and other resources for core operating activities, thereby crowding out investment in computing infrastructure and AI-related projects. Existing studies show that electricity shortages and supply fluctuations can disrupt production decisions and inhibit technology adoption [35,38]. Rising uncertainty also discourages enterprises’ long-term investment, especially in emerging technologies that require substantial investment and involve high risks [39]. Therefore, persistent electricity constraints may limit enterprises’ ability to sustain computing-intensive activities and restrict the scale and depth of AI development.

UHV transmission projects can effectively mitigate the mismatch between regional power supply and demand by enhancing the cross-regional power transmission capabilities [40]. Improved transmission capacity enhances the reliability and accessibility of electricity, reduces enterprises’ exposure to supply disruptions, and enables electricity resources to be allocated more efficiently across regions. These improvements can also reduce energy-related operating burdens and create more favorable conditions for computing-intensive technology investment. Previous research shows that better power supply conditions can improve enterprise productivity and expand the scope for technology adoption [41]. Therefore, enterprises are more able to increase the input of computing power and accelerate the deployment and advancement of AI technology.

Based on the above analysis, we propose the following hypothesis:

**Hypothesis 1.** UHV transmission projects significantly promote enterprises’ AI development

The TOE framework emphasizes that enterprises’ technology adoption and diffusion are jointly shaped by technological, organizational, and environmental contexts [42]. AI development is a resource-intensive and cumulative process that requires sustained technological accumulation, substantial organizational commitment, and continued support from the external environment. Enterprises need sufficient technological capabilities to undertake continuous R&D and model iteration, adequate internal resources to support computing infrastructure, data, and specialized personnel, and favorable external conditions for accessing complementary resources required for long-term technology investment. Energy infrastructure can influence each of these conditions by affecting the stability and accessibility of electricity supply. Therefore, UHV transmission projects may support sustained technological innovation, ease energy-related operating burdens, and create more favorable external conditions for long-term investment. Accordingly, the effects of UHV transmission projects on enterprises’ AI development can be examined from the technological, organizational, and environmental dimensions of the TOE framework. Fig 1 presents the conceptual framework.

**Fig 1:**
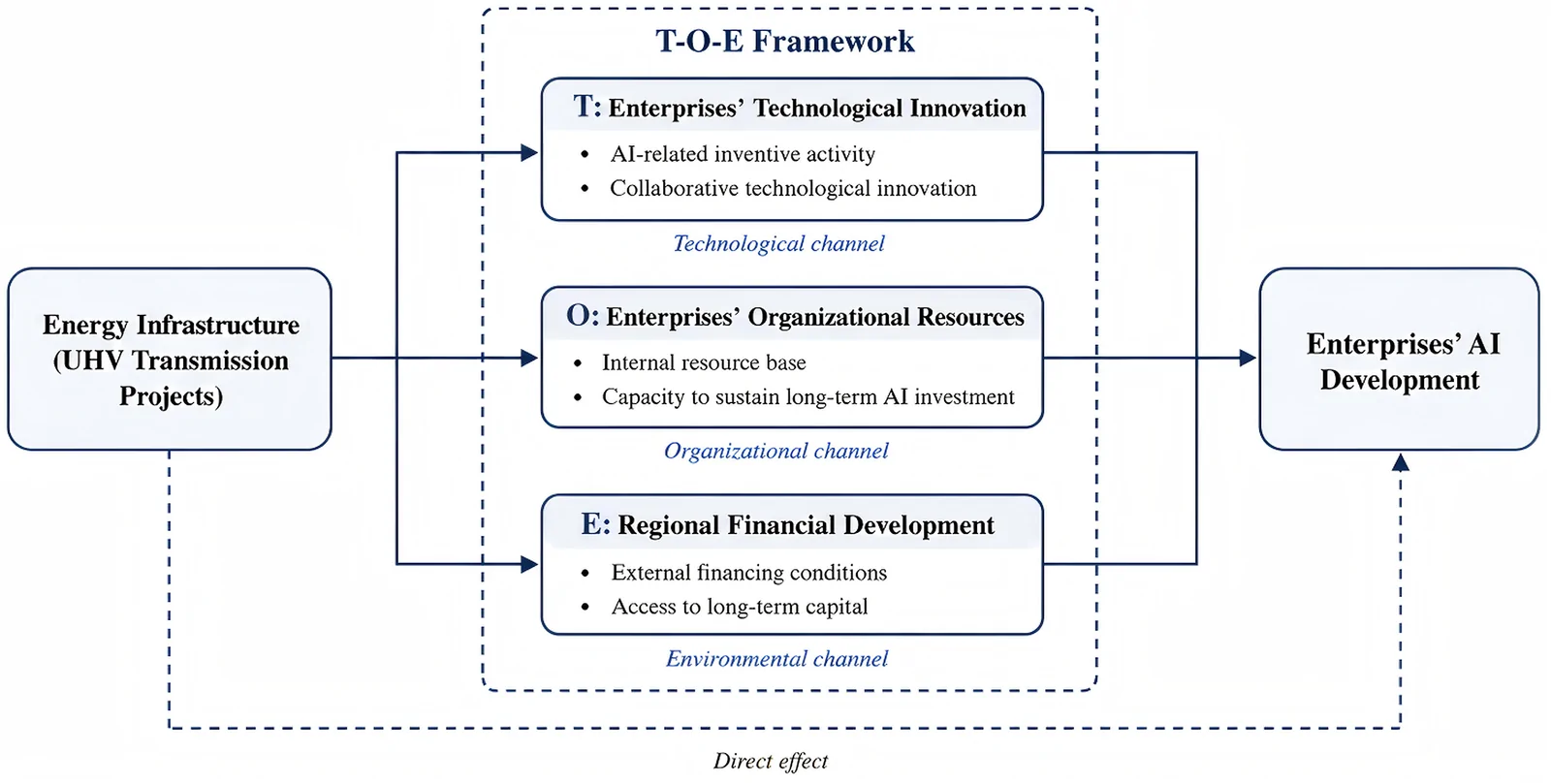
Conceptual framework.

**Technological dimension.** The technological context of the TOE framework concerns the technological capabilities and conditions that enable enterprises to adopt and develop new technologies. As a general-purpose technology, AI relies heavily on continuous knowledge accumulation and sustained R&D capability [43]. Compared with conventional technologies, AI-related R&D is characterized by greater complexity and path dependence, requiring repeated algorithm optimization, model iteration, and long-term technological investment [29]. Under binding energy constraints, enterprises may find it difficult to sustain computing-intensive activities, thereby restricting technological innovation [44]. Previous research shows that improvements in energy supply can reduce uncertainty surrounding R&D activities and improve the predictability of innovation investment, thereby supporting innovation output [45]. By improving power supply stability and reducing the risk of interruptions and voltage fluctuations, UHV transmission projects provide more reliable infrastructure conditions for sustained R&D activities [46]. These improvements can strengthen enterprises’ technological innovation capability and provide a stronger technological foundation for the AI development and application.

**Organizational dimension.** The organizational context of the TOE framework reflects the internal resources and capabilities that determine whether enterprises can effectively adopt emerging technologies. AI development requires substantial initial and continuing investment in computing infrastructure, data resources, specialized personnel, and related organizational capabilities [32]. Consequently, enterprises with limited internal resources may be less able to transform favorable technological conditions into actual AI development. UHV transmission projects can improve enterprises’ operating conditions by reducing energy-related costs and stabilizing electricity supply [11]. More reliable electricity supply reduces production disruptions and the associated operating losses, thereby preserving internal resources that can be allocated to R&D and technological capability building [47,48]. Therefore, UHV transmission projects can strengthen enterprises’ organizational capacity to undertake AI development by relaxing internal resource constraints.

**Environmental dimension.** The environmental context of the TOE framework concerns the external conditions that shape enterprises’ technology adoption and resource acquisition. For AI development, external financing conditions are particularly important because AI-related investment is capital-intensive, long-term, and subject to considerable uncertainty. Therefore, enterprises often require sustained access to external capital in addition to internally generated resources [49,50]. A more developed regional financial system can reduce financing frictions, improve capital allocation efficiency, and facilitate enterprises’ access to long-term funding for innovation and digital transformation [51,52]. By improving cross-regional energy allocation and electricity supply conditions, UHV transmission projects can support regional production and investment activities. More broadly, infrastructure improvements can facilitate resource flows and financial agglomeration, thereby creating more favorable conditions for regional financial development [53]. Improved regional financial development can then enhance enterprises’ access to stable external funding for AI-related R&D, computing investment, and technology deployment, thereby promoting their AI development.

Based on the above analysis, we propose the following hypotheses:

**Hypothesis 2a.** UHV transmission projects promote enterprises’ AI development by strengthening enterprises’ technological innovation capability.

**Hypothesis 2b.** UHV transmission projects promote enterprises’ AI development by expanding enterprises’ internal organizational resources.

**Hypothesis 2c.** UHV transmission projects promote enterprises’ AI development by improving regional financial development.

## 3. Methodology

### 3.1. Model setting

Using panel data of Chinese listed enterprises from 2007–2023, we treat the staggered rollout of UHV transmission projects across prefecture-level cities as a quasi-natural experiment to estimate their effect on enterprises’ AI development. For each city, we identify the year when it first gained access to the UHV network. Enterprises located in cities connected to UHV transmission projects are assigned to the treatment group from the corresponding year onward, while enterprises in cities without UHV access constitute the control group. To avoid capturing partial-year effects, UHV access occurring between July and December is treated as effective in the following year. Given the phased construction and staggered operation of UHV transmission projects across cities, this setting provides an appropriate framework for DID estimation. This identification strategy has been extensively applied in evaluating infrastructure and policy impacts [54,55].

The treatment variable is defined at the prefecture-city level because the effects of UHV transmission projects are realized through regional power systems rather than directly targeting individual enterprises. Therefore, the connection of a city to the UHV network represents an exogenous change in the external electricity supply environment faced by local enterprises. Based on this identification strategy, we construct the following baseline model:


$$\begin{array}{c} {AI}_{it}=\beta_{\text{0}}+\beta_{\text{1}}{UHV}_{it}+\beta_{m}{Controls}_{it}+{Year}_{t}+{Company}_{i}+{\text{ℇ}}_{it} \end{array}$$ (1)

where *i* denotes enterprises and *t* denotes years. *AI* denotes enterprises’ AI development. *UHV* denotes the dummy variable of energy infrastructure construction. *Controls* denotes a set of control variables at the enterprise and city levels. *Year* and *Company* denote the year and enterprise fixed effects, respectively. $\begin{array}{c} {\text{ ℇ}}_{it} \end{array}$ denotes the random error term. Standard errors are clustered at the city level to address potential issues of serial correlation.

### 3.2. Variable definitions

**Explained variable (AI).** Following prior studies that use corporate disclosures to capture enterprises’ engagement with AI [56–58], we construct a text-based measure of enterprises’ AI development from their annual reports. Specifically, we compile an expanded dictionary of AI-related terms and count the total number of occurrences of these keywords in each firm-year annual report. Because the resulting count variable is highly right-skewed and contains zero values, we define AI as the natural logarithm of one plus the total keyword frequency. Annual reports contain information on enterprises’ strategic priorities, technological activities, and operating plans, making textual disclosures useful for identifying their engagement with emerging technologies. Compared with patent-based measures, the textual indicator can also capture AI-related activities at an earlier stage, before they are reflected in observable patent outcomes. Nevertheless, as textual measures may be affected by differences in disclosure intensity and report content, we conduct additional robustness tests using alternative specifications of the AI indicator.

**Key explanatory variable (UHV).** We use official records from the National Energy Administration, State Grid Corporation of China, and China Southern Power Grid to identify the commissioning date and route of each UHV transmission line. A firm-year is coded as 1 from the year its region first gains access to the UHV network onward, and 0 otherwise. In defining UHV access, we distinguish between DC and AC lines because their spatial transmission structures differ. DC lines are typically point-to-point without intermediate nodes, so the endpoint regions are treated as beneficiaries. AC lines may include intermediate substations, and regions along the route with such substations are therefore also classified as beneficiaries. This approach better matches the spatial and technical features of UHV transmission projects and helps identify where electricity supply conditions are likely to improve in practice [59].

**Control variables.** Referring to existing studies [60], we incorporate a series of firm-level and city-level control variables to mitigate potential omitted variable bias. Firm-level controls include: enterprise size (Size), the natural logarithm of operating revenue; enterprise age (Age), the natural logarithm of years since listing; cash flow (CF), the proportion of net cash flow from operating activities in total assets; fixed assets intensity (Far), the proportion of net fixed assets in total assets; growth (Growth), annual revenue growth rate; board size (Board), the natural logarithm of directors; ownership concentration (Top1), shareholding of the largest shareholder; female director ratio (Female), the proportion of female directors in the total number of board directors; and audit opinion (Opinion), a dummy that equals 1 for enterprise receiving a standard unqualified audit opinion and 0 otherwise. City-level controls include: fiscal self-sufficiency rate (Finance), the proportion of local general budgetary revenue in local general budgetary expenditure; and science and technology development level (Tech), the proportion of science and technology expenditure in local general budgetary expenditure.

### 3.3. Data and descriptive statistics

The empirical analysis draws on the panel data of Chinese A-share listed enterprises from 2007 to 2023. Annual reports are retrieved from Sina Finance platform. Firm-level financial data and basic corporate information are sourced from the China Stock Market & Accounting Research (CSMAR) Database and the China Research Data Services (CNRDS) Platform. City-level data are obtained from the China Statistical Yearbook.

Sample construction follows the procedures below. First, financial and insurance enterprises are excluded, as their balance sheets are different in nature and the comparability of innovation activities is limited. Second, eliminate the enterprises that are designated as ST or *ST during the sample period. Third, observations with missing values for core variables are removed from the dataset. Finally, to limit the interference of extreme values, all continuous variables are winsorized at the 1% and 99% percentiles within each year. To guarantee data integrity and structural consistency, we apply linear interpolation when necessary. Descriptive statistics for the main variables are reported in Table 1.

**Table 1:** Descriptive statistics.

Variables	Obs	Mean	SD	Min	Max
AI	40,450	0.898	1.234	0	4.745
UHV	40,450	0.321	0.467	0	1
Size	40,450	21.561	1.448	17.863	26.011
Age	40,450	2.185	0.764	0.693	3.434
CF	40,450	0.050	0.069	−0.235	0.308
Far	40,450	0.217	0.161	0.002	0.784
Growth	40,450	0.151	0.355	−0.648	3.347
Board	40,450	2.127	0.199	1.609	2.708
Top1	40,450	34.211	14.893	8.380	74.020
Female	40,450	0.150	0.133	0	0.800
Opinion	40,450	0.978	0.148	0	1
Finance	40,450	0.728	0.205	0.142	1.541
Tech	40,450	0.043	0.027	0.002	0.178

## 4. Empirical results

### 4.1. Baseline results

Table 2 presents the baseline regression results of Model (1). Column (1) includes only the UHV indicator, whose coefficient is positive and significant at the 1% level. Columns (2) to (4) sequentially add corporate financial controls, corporate governance controls, and city-level controls. The UHV coefficient remains significantly positive and statistically significant across all specifications, indicating that the association between UHV access and enterprises’ AI development is not driven by these observable firm- or city-level characteristics.

**Table 2:** Baseline results.

Variables	(1)	(2)	(3)	(4)
	AI	AI	AI	AI
UHV	0.109***	0.103***	0.100***	0.098***
	(0.042)	(0.038)	(0.038)	(0.037)
Size		0.163***	0.163***	0.161***
		(0.020)	(0.021)	(0.020)
Age		0.192***	0.167***	0.157***
		(0.036)	(0.038)	(0.038)
CF		−0.293***	−0.294***	−0.294***
		(0.060)	(0.059)	(0.058)
Far		−0.437***	−0.426***	−0.421***
		(0.083)	(0.083)	(0.084)
Growth		−0.049***	−0.046***	−0.044***
		(0.012)	(0.011)	(0.011)
Board			0.185***	0.185***
			(0.052)	(0.052)
Top1			−0.004***	−0.005***
			(0.001)	(0.001)
Female			−0.031	−0.029
			(0.064)	(0.064)
Opinion			0.044	0.045
			(0.038)	(0.038)
Finance				−0.281***
				(0.082)
Tech				2.125***
				(0.727)
Year	Yes	Yes	Yes	Yes
Company	Yes	Yes	Yes	Yes
Constant	0.863***	−2.960***	−3.173***	−2.994***
	(0.013)	(0.466)	(0.489)	(0.483)
N	40,450	40,450	40,450	40,450
Adj.R^2^	0.763	0.770	0.771	0.772

Notes:***, **, and *, respectively, indicate that the coefficients are significant at the levels of 1%, 5%, and 10%. Standard errors in parentheses are clustered at the city level.

In Column (4), the estimated coefficient of UHV is 0.098, indicating an economically meaningful improvement in enterprises’ AI development following UHV access. More broadly, this result suggests that AI development depends not only on enterprises’ internal capabilities and policy support, but also on the infrastructure conditions under which enterprises operate. By strengthening cross-regional power transmission, UHV transmission projects may provide a more supportive energy environment for enterprises to undertake electricity- and computing-intensive AI activities. Overall, the baseline results provide strong statistical and economic support for Hypothesis 1.

### 4.2. Parallel trend test

A key prerequisite for identifying the treatment effect using a multi-period DID model is the parallel trends assumption [61]. Considering the relatively limited sample support at event times distant from policy implementation, we set the event-study window to five years before and after UHV access, with the year immediately preceding treatment serving as the reference period following prior research [62]. This window provides a sufficiently long pre-treatment period for assessing trend comparability while allowing the dynamic post-treatment effects to be captured.

As shown in Fig 2, the estimated coefficients in the pre-treatment periods remain close to zero and are statistically indistinguishable from zero. A further joint significance test fails to reject the null hypothesis that all pre-treatment coefficients are jointly equal to zero (p = 0.1386). This suggests that there is no significant systematic difference in the pre-treatment trends of AI development between firms in the treatment and control groups prior to UHV access, providing empirical support for the parallel trends assumption.

**Fig 2:**
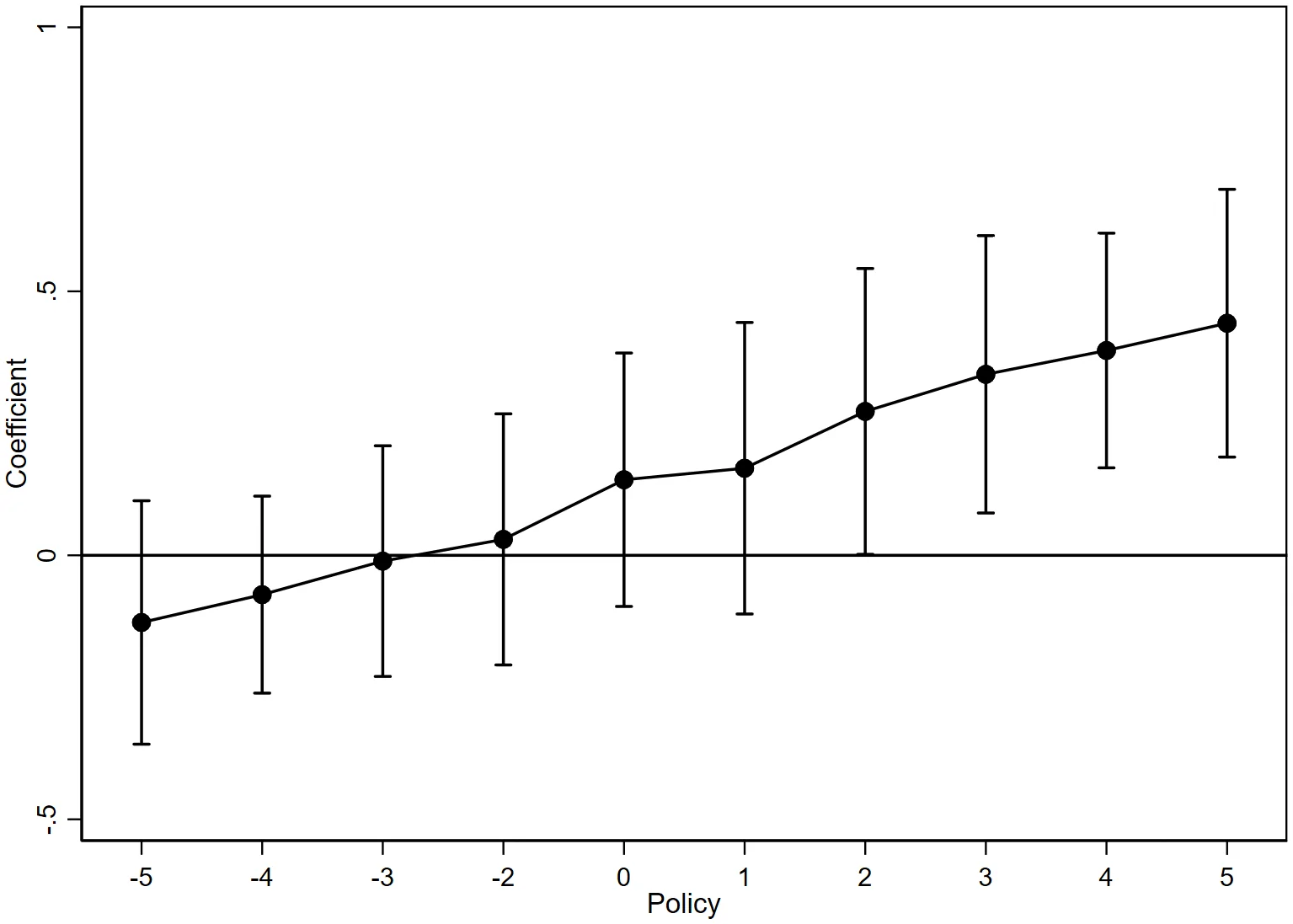
Parallel trend test results.

Following UHV access, the estimated coefficients become positive and exhibit a generally increasing pattern over time, indicating that the effect of UHV transmission projects on enterprises’ AI development is persistent and cumulative. The treatment effect does not fully materialize immediately after UHV access but becomes stronger in subsequent periods. One possible explanation is that improvements in electricity infrastructure require time to translate into innovation outcomes through adjustments in computing-resource allocation, digital investment, and R&D activities. Therefore, the innovation-enhancing effect of improved electricity infrastructure may be gradually realized over time. Overall, these findings not only provide empirical support for the parallel trends assumption underlying the DID model but also reveal the dynamic nature of the effect of UHV transmission projects on enterprises’ AI development.

### 4.3. Placebo test

To assess whether the baseline results are driven by unobserved confounders, omitted variables, or other concurrent factors, we conduct a placebo test following Autor and Dorn [63]. Specifically, we randomly assign a UHV commissioning year to each sample city and construct a placebo treatment indicator (UHV_false). We then re-estimate the baseline specification 1,000 times using the randomly assigned treatment and obtain the corresponding distributions of the estimated coefficients and P-values. Under a valid identification strategy, the coefficients generated from these placebo treatments should be concentrated around zero and exhibit little systematic statistical significance. Fig 3 reports the results.

**Fig 3:**
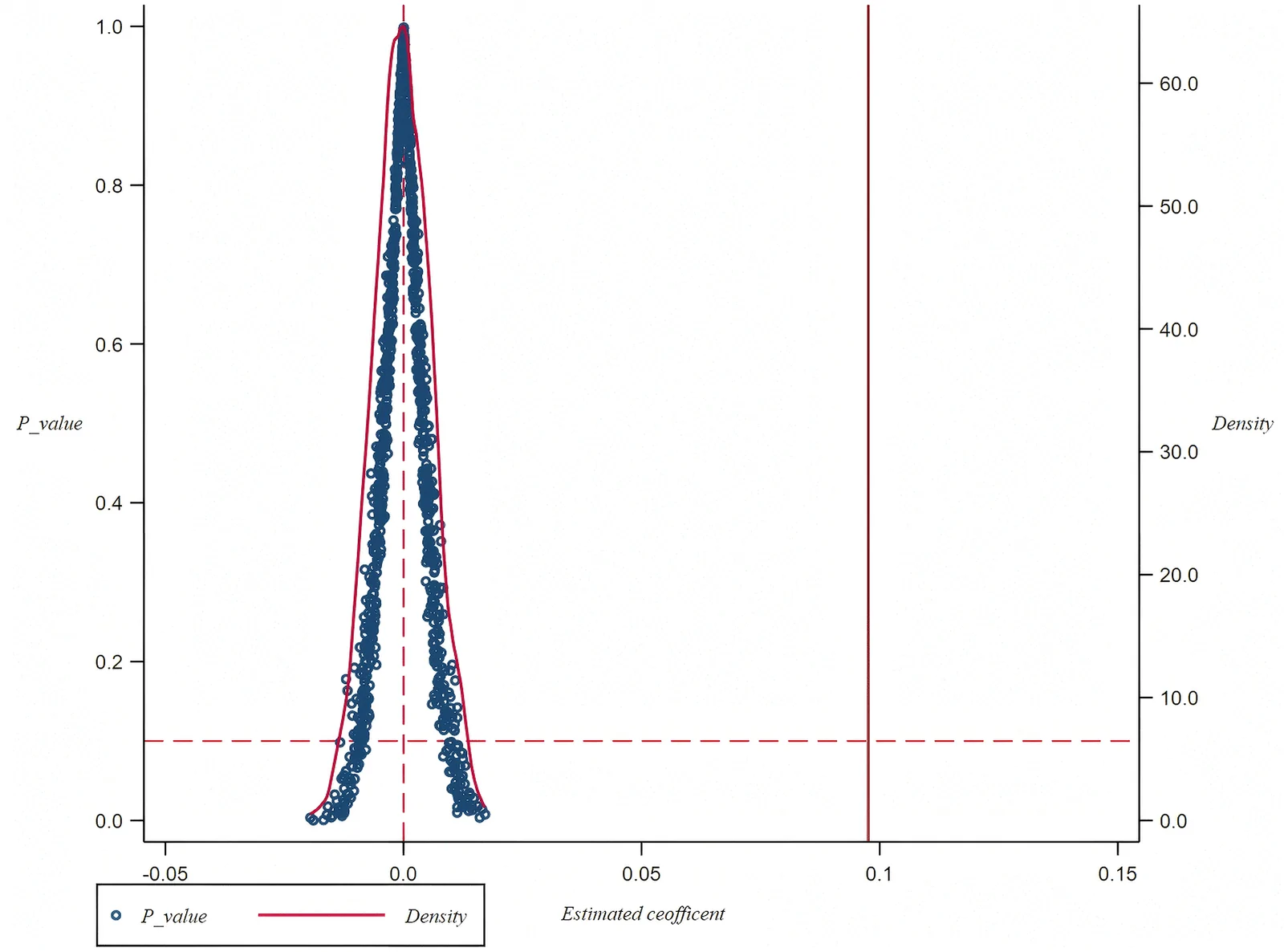
Placebo test results.

The placebo coefficients are largely concentrated around zero, and most estimates are statistically insignificant. In contrast, the baseline coefficient of 0.098 lies in the right tail of the placebo distribution and is substantially larger than most simulated estimates. These findings suggest that the baseline effect is unlikely to be explained by random treatment assignment or other unobserved factors. Instead, the estimated effect is more likely to reflect systematic changes associated with access to UHV transmission projects rather than incidental variations across cities and years. Overall, the placebo test provides further support for the robustness of the baseline results.

### 4.4. Robustness checks

#### 4.4.1. Instrumental variable strategy.

To address potential endogeneity arising from the non-random placement of UHV transmission projects, we further apply an IV approach to retest Hypothesis 1. Following Chen and Cheng [2], we use the interaction between the logarithm of the number of national nature reserves in each province and the post-operation indicator (Protect×Post) as the instrument. On the one hand, the instrument satisfies correlation, as the UHV routes are deliberately planned to bypass national nature reserves during the site selection process. On the other hand, it satisfies exogenousness, as natural reserves are areas designated by law for special protection and management, and do not directly affect enterprises’ AI development.

Table 3 reports the two-stage least squares (2SLS) results. The first-stage estimate in Column (1) shows a significant relationship between the instrument and UHV transmission projects. The Kleibergen-Paap Wald rk F statistic is 196.19, well above the Stock-Yogo critical value of 16.38, indicating strong instrument relevance. In the second stage in Column (2), the coefficient of UHV remains significantly positive at the 5% level. The Kleibergen-Paap rk LM statistic is 21.43 (p = 0.000), rejecting under-identification. These results suggest that the positive effect of UHV transmission projects is unlikely to be driven solely by endogenous project placement. More broadly, they indicate that the role of UHV transmission projects in enterprises’ AI development is not merely a reflection of projects being located in regions already better positioned for AI adoption, but is associated with the change in external infrastructure conditions itself. Therefore, the main conclusion remains robust.

**Table 3:** Instrumental variable strategy results.

Variables	(1)	(2)
	Phase I	Phase II
	UHV	AI
Protect×Post	0.415***	
	(0.030)	
UHV		0.100**
		(0.048)
Controls	Yes	Yes
Year	Yes	Yes
Company	Yes	Yes
N	40,450	40,450
Adj.R^2^	0.948	0.038
Kleibergen-Paap Wald rk F statistic	196.19***	
	{16.38}	
Kleibergen-Paap rk LM statistic	21.43	
	[0.000]	

Notes: ***, **, and *, respectively, indicate that the coefficients are significant at the levels of 1%, 5%, and 10%. Standard errors in parentheses are clustered at the city level. P-value is in square brackets. Critical value for the Stock-Yogo weak identification test at the 10% level is in curly braces.

#### 4.4.2. Propensity score matching.

To reduce potential selection bias, we employ a propensity score matching and difference-in-differences (PSM-DID) approach. Specifically, Size, Age, CF, Board, Top1, Female, and Opinion in Model (1) are used as matching covariates, and UHV serves as the treatment indicator. The sample enterprises in the treatment group and in the control group are matched at a ratio of 1:2. After matching, the absolute standardized biases of all covariates fall below 10%, indicating satisfactory balance between the two groups. Column (1) of Table 4 reports the baseline regression results after sample matching. The coefficient of UHV remains significantly positive at the 1% level. This result suggests that the baseline effect is not primarily driven by systematic differences in observable characteristics between treated and untreated enterprises. It also strengthens the interpretation that the observed change in AI development is associated with UHV access rather than pre-existing sample composition.

**Table 4:** PSM-DID and alternative dependent variable results.

Variables	(1)	(2)	(3)
	PSM-DID	Alternative dependent variable	
	AI	AI_Intensity	AI_MD&A
UHV	0.112***	0.078*	0.090***
	(0.042)	(0.045)	(0.028)
Controls	Yes	Yes	Yes
Year	Yes	Yes	Yes
Company	Yes	Yes	Yes
Constant	−2.951***	−1.134***	−2.344***
	(0.514)	(0.221)	(0.449)
N	31,715	40,450	40,450
Adj.R^2^	0.768	0.767	0.736

Notes:***, **, and *, respectively, indicate that the coefficients are significant at the levels of 1%, 5%, and 10%. Standard errors in parentheses are clustered at the city level.

#### 4.4.3. Alternative dependent variable.

Text-based measures based on raw keyword counts may be affected by differences in annual-report length, as longer reports mechanically provide more opportunities for AI-related terms to appear. To mitigate this concern, following Mishra et al. [56], we normalize the frequency of AI-related keywords by the total number of words in each annual report and construct AI_Intensity, measured as the number of AI-related keywords per 10,000 words. This specification captures the relative intensity of AI-related disclosure rather than its absolute frequency. Column (2) of Table 4 shows that the coefficient of UHV remains significantly positive, indicating that the baseline result is not driven by differences in report length or disclosure verbosity.

We further address the possibility that AI-related terms appearing in other sections of annual reports may reflect general disclosure rather than firm-specific technological activities. The Management Discussion and Analysis (MD&A) section contains management’s discussion of operating conditions and forward-looking information and has been widely used in textual research to extract economically meaningful firm-level information [64]. We therefore reconstruct the dependent variable using only the MD&A section and define AI_MD&A as the natural logarithm of one plus the frequency of AI-related keywords in this section. Column (3) of Table 4 shows that the coefficient of UHV remains significantly positive. Because the MD&A provides a more focused account of enterprises’ operations and future plans, the persistence of the UHV effect under this narrower textual measure reduces the concern that the baseline finding is driven by AI-related language appearing in less relevant sections of annual reports.

Taken together, these two alternative measures address different sources of potential measurement error in the text-based AI indicator. Normalizing by report length mitigates mechanical differences arising from disclosure volume, while restricting the analysis to the MD&A reduces noise from less relevant sections of annual reports. The consistent results provide stronger evidence that the baseline AI measure captures variation in enterprises’ AI-related strategic and technological engagement rather than merely differences in reporting style or document structure.

#### 4.4.4. Excluding concurrent policies.

Several national policies implemented during the sample period may also affect enterprises’ AI development. To account for these concurrent policy shocks, we further control for three major initiatives. First, we consider the National Big Data Comprehensive Pilot Zone (NBDPZ) policy, which was launched in 2015 and subsequently expanded in batches, and construct DID1 accordingly (Table 5, Column (1)). Second, we control for the Broadband China (BC) policy, introduced in 2013 and gradually extended to additional pilot cities during 2014–2016, using DID2 (Table 5 Column (2)). Third, we consider the National Artificial Intelligence Innovation and Development Pilot Zone (NAIPZ) policy, implemented in three batches between 2019 and 2021, and construct DID3 (Table 5, Column (3)). Each indicator equals 1 from the year in which the corresponding policy was implemented in the enterprise’s city and 0 otherwise.

**Table 5:** Excluding concurrent policies results.

Variables	(1)	(2)	(3)
	NBDPZ	BC	NAIPZ
	AI	AI	AI
UHV	0.077**	0.084**	0.076**
	(0.031)	(0.037)	(0.036)
DID1	0.101**		
	(0.045)		
DID2		0.121***	
		(0.039)	
DID3			0.108***
			(0.030)
Controls	Yes	Yes	Yes
Year	Yes	Yes	Yes
Company	Yes	Yes	Yes
Constant	−3.011***	−3.055***	−2.982***
	(0.475)	(0.484)	(0.476)
N	40,450	40,450	40,450
Adj.R^2^	0.772	0.772	0.772

Notes:***, **, and *, respectively, indicate that the coefficients are significant at the levels of 1%, 5%, and 10%. Standard errors in parentheses are clustered at the city level.

Across all specifications, the coefficient of UHV remains positive and statistically significant. Notably, the three policy variables themselves are also positively significant, indicating that digital infrastructure and AI-oriented policies contribute to enterprises’ AI development, while the persistent UHV effect points to energy infrastructure as an additional external condition supporting this process.

#### 4.4.5. Additional robustness checks.

We conduct a set of additional robustness checks across four dimensions to further verify the reliability of our findings. First, to account for time-varying industry-level shocks that may affect enterprises’ AI development, we include industry-by-year fixed effects (Ind×Year). The result is reported in Column (1) of Table 6. Second, the four municipalities (Beijing, Shanghai, Tianjin, and Chongqing) are administratively comparable to provinces and differ markedly from other cities in market size, development level, and policy environment. They may therefore influence the estimates. We re-estimate the model after excluding enterprises located in these municipalities. The result is shown in Column (2) of Table 6. Third, because the COVID-19 pandemic represents a major exogenous shock to enterprise operations and technology investment, we exclude the observations during the epidemic period from 2020 to 2022 for re-regression. The result is shown in Column (3) of Table 6. Finally, we further examine the temporal persistence of the effect of UHV transmission projects by using enterprises’ AI development in the subsequent period as the dependent variable. This specification provides an additional robustness check on the relationship between UHV transmission projects and enterprises’ AI development. The result is reported in Column (4) of Table 6.

**Table 6:** Additional robustness checks.

Variables	(1)	(2)	(3)	(4)
	Ind×Year FE	Excl. Muni	Excl. Covid	Lagged explained variable
	AI	AI	AI	L_AI
UHV	0.060*	0.092*	0.086***	0.103**
	(0.032)	(0.053)	(0.030)	(0.041)
Controls	Yes	Yes	Yes	Yes
Year	Yes	Yes	Yes	Yes
Company	Yes	Yes	Yes	Yes
Ind×Year	Yes	No	No	No
Constant	−2.603***	−3.388***	−3.094***	−2.987***
	(0.444)	(0.529)	(0.487)	(0.526)
N	40,436	32,256	28,496	35,320
Adj.R^2^	0.790	0.758	0.728	0.763

Notes:***, **, and *, respectively, indicate that the coefficients are significant at the levels of 1%, 5%, and 10%. Standard errors in parentheses are clustered at the city level.

Across these alternative specifications, the coefficient of UHV remains significantly positive. The consistency of the estimates indicates that the baseline finding is not driven by industry-specific shocks, the inclusion of municipalities, or pandemic-related disruptions. Moreover, the result using the lagged AI measure provides additional support for the robustness of the baseline finding. Taken together, these tests further strengthen the reliability of the estimated effect of UHV transmission projects on enterprises’ AI development.

### 4.5. Heterogeneity analyses

The effect of UHV transmission projects on enterprises’ AI development may vary with enterprises’ initial constraints and their capacity to convert improved infrastructure conditions into technology investment. On the one hand, the marginal value of electricity infrastructure is likely to be greater for enterprises facing stronger resource or infrastructure constraints, because improvements in electricity availability can relax previously binding limitations. On the other hand, the extent to which these benefits translate into AI development depends on enterprises’ organizational responsiveness and technological absorptive capacity. Accordingly, we examine heterogeneity along four dimensions: ownership, regional location, corporate governance structure, and technological intensity. The first two dimensions capture differences in pre-existing constraints, whereas the latter two reflect differences in enterprises’ ability to respond to and utilize improved infrastructure conditions.

#### 4.5.1. Ownership type.

Ownership differences may shape enterprises’ exposure to external resource constraints. Compared with state-owned enterprises (SOEs), non-SOEs generally have weaker access to financing and other strategic resources, making long-term and uncertain AI investment more sensitive to changes in operating conditions. Therefore, improvements in electricity availability and reliability may generate greater marginal benefits for non-SOEs, whereas stronger resource access may reduce SOEs’ responsiveness to the same infrastructure improvement. We thus expect the effect of UHV transmission projects on AI development to be stronger for non-SOEs.

To test this expectation, we construct a binary indicator (SOE), which equals 1 for SOEs and 0 for non-SOEs, and divide the sample accordingly. Columns (1)-(2) of Table 7 show that the coefficients of UHV are positive in both groups, but statistically significant only for non-SOEs. The coefficient difference test further confirms that the effect is significantly larger for non-SOEs. This finding suggests that energy infrastructure generates greater marginal value for enterprises facing stronger initial resource constraints.

**Table 7:** Heterogeneity analysis results.

Variables	(1)	(2)	(3)	(4)	(5)	(6)	(7)	(8)
	SOEs	non-SOEs	Coastal	Inland	Dual	non-Dual	High-tech	non-High-tech
	AI	AI	AI	AI	AI	AI	AI	AI
UHV	0.062	0.132**	0.016	0.145***	0.137**	0.067**	0.173**	0.074**
	(0.043)	(0.062)	(0.059)	(0.037)	(0.068)	(0.033)	(0.078)	(0.034)
Controls	Yes	Yes	Yes	Yes	Yes	Yes	Yes	Yes
Year	Yes	Yes	Yes	Yes	Yes	Yes	Yes	Yes
Company	Yes	Yes	Yes	Yes	Yes	Yes	Yes	Yes
Constant	−1.924***	−3.216***	−3.613***	−2.487***	−3.254***	−2.621***	−2.784***	−3.118***
	(0.567)	(0.586)	(0.797)	(0.459)	(0.858)	(0.503)	(0.786)	(0.531)
N	15,955	24,430	16,781	23,655	10,571	29,371	10,548	29,784
Adj.R^2^	0.698	0.795	0.775	0.768	0.825	0.758	0.804	0.766
Difference in coefficient	0.070**		0.129***		−0.070**		−0.099***	

Notes:***, **, and *, respectively, indicate that the coefficients are significant at the levels of 1%, 5%, and 10%. Standard errors in parentheses are clustered at the city level. Differences in coefficient are obtained by adopting the bootstrap-based Fisher permutation test with 1000 repeated sampling replications.

#### 4.5.2. Regional heterogeneity.

The marginal value of infrastructure improvement may also depend on existing regional infrastructure conditions. Coastal regions generally have more developed infrastructure, factor markets, and innovation ecosystems, leaving relatively less room for additional energy infrastructure to relax existing constraints. In contrast, enterprises in inland regions are more likely to face limitations in infrastructure accessibility and factor allocation, making improvements in electricity supply particularly valuable for electricity- and computing-intensive activities. Therefore, we expect the effect of UHV transmission projects on enterprises’ AI development to be stronger in inland regions.

To examine regional differences, we construct a binary indicator (Coastal), which equals 1 for enterprises located in coastal regions and 0 for those in inland regions. Columns (3)-(4) of Table 7 show that the coefficients of UHV are positive in both groups, but statistically significant only for inland enterprises. The coefficient difference test confirms that the effect is significantly larger in inland regions. This result indicates that infrastructure investment can generate larger marginal returns where pre-existing infrastructure constraints are more binding.

#### 4.5.3. Corporate governance structure heterogeneity.

The benefits of improved infrastructure may depend not only on external conditions but also on enterprises’ ability to respond to them. A more concentrated decision-making structure can reduce internal coordination costs and facilitate the rapid allocation of financial, technological, and human resources when external operating conditions improve. CEO-chair duality may therefore enable enterprises to translate improved electricity conditions into AI-related investment more rapidly, whereas separated leadership may involve greater coordination in resource adjustment. Thus, we expect the effect of UHV transmission projects to be stronger for enterprises with CEO-chair duality.

To examine this possibility, we construct a binary indicator (Dual), which equals 1 when the chairman and general manager positions are held by the same person and 0 otherwise. Columns (5)-(6) of Table 7 show that UHV transmission projects significantly promote enterprises’ AI development in both groups, while the coefficient difference test indicates a significantly larger effect for enterprises with CEO-chair duality. This finding suggests that enterprises’ organizational responsiveness affects their ability to convert infrastructure improvements into AI-related investment.

#### 4.5.4. Industry heterogeneity.

The effect of energy infrastructure may further depend on enterprises’ technological intensity and absorptive capacity. High-tech enterprises generally have stronger digital foundations, greater demand for computing-intensive R&D, and greater capacity to integrate new technologies into production and innovation activities. Therefore, improved electricity availability and reliability can be more readily converted into AI investment and application in these enterprises. By contrast, enterprises with lower dependence on computing-intensive technologies may derive smaller marginal gains from the same infrastructure improvement. Thus, we expect the effect of UHV transmission projects on AI development to be stronger for high-tech enterprises.

To test this expectation, we construct a binary indicator (High-tech), which equals 1 for high-tech enterprises and 0 otherwise. Following the Classification of High-tech Manufacturing Industries issued by the National Bureau of Statistics of China (2017) and the revised Industry Classification Guidelines for Listed Companies issued by the China Securities Regulatory Commission (2012), six relevant industries are classified as high-tech industries. Columns (7)-(8) of Table 7 show that UHV transmission projects significantly promote enterprises’ AI development in both groups, with the coefficient difference test indicating a significantly larger effect for high-tech enterprises. The result supports the view that energy infrastructure creates greater value when it is complemented by stronger technological absorption and application capabilities.

Overall, the heterogeneity results support the two theoretical considerations outlined above. The marginal benefits of energy infrastructure are greater where pre-existing resource or infrastructure constraints are more binding, as reflected in the stronger effects among non-SOEs and inland enterprises. At the same time, the conversion of infrastructure improvements into AI development depends on enterprises’ internal capabilities, with stronger effects observed among enterprises with more responsive governance structures and greater technological intensity. These findings suggest that the value of energy infrastructure is jointly shaped by constraint relief and capability complementarity.

## 5. Further analyses

### 5.1. Mechanism tests

Building on the theoretical analysis, we further examine the channels through which UHV transmission projects affect enterprises’ AI development from the technological, organizational, and environmental dimensions of the TOE framework. Specifically, we consider technological innovation capability, organizational resource base, and regional financial development. Following the approach of Baron and Kenny (1986) [65], we examine the proposed mechanisms by estimating the effects of UHV transmission projects on the corresponding mechanism variables and then incorporating these variables into the baseline specification. The mechanism models are specified as follows:


$$\begin{array}{c} M_{it}=\alpha_{\text{0}}+\alpha_{\text{1}}{UHV}_{it}+\alpha_{m}{Controls}_{it}+{Year}_{t}+{Company}_{i}+{\text{ℇ}}_{it} \end{array}$$ (2)

$$\begin{array}{c} {AI}_{it}=\alpha_{\text{0}}+\alpha_{\text{1}}{UHV}_{it}+{\alpha_{\text{2}}M}_{it}+\alpha_{m}{Controls}_{it}+{Year}_{t}+{Company}_{i}+{\text{ℇ}}_{it} \end{array}$$ (3)

where, *M* represents a mechanism variable, and the other parameters are consistent with Model (1).

#### 5.1.1. Technological dimension.

Reliable electricity supply provides an important infrastructure condition for sustained R&D and computing-intensive innovation. We therefore examine whether UHV transmission projects promote enterprises’ AI development by strengthening technological innovation. Following previous studies [66,67], we employ two measures of technological innovation: the number of AI-related invention patent applications (Patent_AI) and the number of jointly applied green utility model patents (Patent_Green), which capture innovation quality and the sustainability of innovation activities, respectively.

Columns (1) and (3) of Table 8 show that the coefficients of UHV are significantly positive at the 10% and 1% levels, respectively. This indicates that UHV transmission projects are associated with greater AI-related inventive activity and collaborative technological innovation. In Columns (2) and (4), the coefficients of Patent_AI and Patent_Green are both significantly positive at the 1% level, while the coefficient of UHV remains significantly positive.

**Table 8:** Mechanism test results.

Variables	(1)	(2)	(3)	(4)	(5)	(6)	(7)	(8)
	T				O		E	
	Patent_AI	AI	Patent_Green	AI	Org_Size	AI	City_Fin	AI
UHV	0.036*	0.093**	0.035***	0.095**	0.024**	0.094**	0.216***	0.078**
	(0.019)	(0.036)	(0.012)	(0.037)	(0.012)	(0.037)	(0.076)	(0.034)
Patent_AI		0.122***						
		(0.015)						
Patent_Green				0.061***				
				(0.018)				
Org_Size						0.149***		
						(0.028)		
City_Fin								0.089***
								(0.029)
Controls	Yes	Yes	Yes	Yes	Yes	Yes	Yes	Yes
Year	Yes	Yes	Yes	Yes	Yes	Yes	Yes	Yes
Company	Yes	Yes	Yes	Yes	Yes	Yes	Yes	Yes
Constant	−0.751***	−2.902***	−0.885***	−2.939***	7.549***	−4.118***	1.864***	−3.159***
	(0.214)	(0.471)	(0.136)	(0.483)	(0.298)	(0.451)	(0.274)	(0.498)
N	40,450	40,450	40,450	40,450	40,450	40,450	40,450	40,450
Adj.R^2^	0.753	0.774	0.505	0.772	0.956	0.773	0.941	0.772

Notes:***, **, and *, respectively, indicate that the coefficients are significant at the levels of 1%, 5%, and 10%. Standard errors in parentheses are clustered at the city level.

These findings suggest that improved electricity infrastructure contributes to the technological accumulation underlying enterprises’ AI development. Stable electricity supply not only supports current production but also creates more favorable conditions for continuous R&D, experimentation, and technology iteration. This is particularly important for AI, whose development depends on cumulative technological learning rather than one-off investment. The results therefore provide support for the technological channel proposed in Hypothesis 2a.

#### 5.1.2. Organizational dimension.

AI development requires substantial internal resources for computing infrastructure, data accumulation, specialized personnel, and long-term R&D. Improvements in electricity supply can reduce energy-related operating burdens and production disruptions, thereby preserving resources that enterprises can allocate to technology investment. Therefore, we examine whether UHV transmission projects promote AI development by expanding enterprises’ organizational resource base. Following Arbelo et al. [68], we use organizational resource capacity (Org_Size), measured as the natural logarithm of total assets, to capture the overall resources available within the enterprise to support long-term investment.

Column (5) of Table 8 shows that the coefficient of UHV is significantly positive at the 5% level, suggesting that UHV transmission projects contribute to the expansion of enterprises’ internal resource base. When Org_Size is included in the AI regression in Column (6), its coefficient is significantly positive at the 1% level, while the coefficient of UHV remains significantly positive.

These results indicate that the benefits of improved energy infrastructure extend beyond the direct use of electricity. By reducing production disruptions and energy-related operating burdens, UHV transmission projects can help enterprises preserve and accumulate internal resources. Such resource accumulation is important for AI development because computing infrastructure, specialized personnel, and long-cycle R&D require substantial upfront commitments before technological returns can be realized. Therefore, a stronger internal resource base enhances enterprises’ capacity to sustain AI investment and absorb the associated risks, providing support for Hypothesis 2b.

#### 5.1.3. Environmental dimension.

AI development also depends on external conditions that shape enterprises’ access to long-term financing. A more developed regional financial system can reduce financing frictions and facilitate access to external capital, thereby providing greater support for capital-intensive and long-cycle technology investment. Therefore, we examine regional financial development as an external financing channel. Following Zhang et al. [69], we measure city-level financial development (City_Fin) as the ratio of year-end deposits of financial institutions to regional GDP. A higher value indicates a greater availability of financial resources relative to the size of the local economy.

Column (7) of Table 8 shows that the coefficient of UHV is significantly positive at the 1% level, indicating that UHV transmission projects are associated with higher regional financial development. In Column (8), the coefficient of City_Fin is significantly positive at the 1% level, while the coefficient of UHV remains significantly positive.

These findings suggest that the influence of energy infrastructure can extend beyond enterprises’ internal operations to the broader external resource environment. By supporting regional production and investment activities, improvements in electricity supply may create more favorable conditions for the allocation of financial resources. Greater regional financial development, in turn, facilitates enterprises’ access to external capital required for AI-related R&D, computing investment, and technology deployment. This is particularly relevant to AI because large and uncertain technology investments may be difficult to sustain solely through internally generated resources. Therefore, the results provide support for the environmental channel proposed in Hypothesis 2c.

### 5.2. Misallocation correction effect

Constraints in traditional electricity transmission can create spatial frictions in energy allocation and further affect enterprises’ allocation of innovation resources. We consider two dimensions of such distortions. First, unstable energy supply may interact with financing constraints and limit enterprises’ ability to sustain large-scale and long-term AI investment, resulting in scale-related resource allocation distortions. Second, under greater operating and financial pressure, enterprises may prioritize resources for short-term production rather than long-term innovation, creating a structural imbalance in resource allocation [70]. By improving cross-regional electricity allocation and supply stability, UHV transmission projects may help alleviate these distortions.

For the scale dimension, we use the absolute value of the SA index from the CSMAR database to measure financing constraints, with a higher value indicating tighter constraints. Enterprises are divided into high- and low-financing-constraint groups using the industry-year median. Columns (1)-(2) of Table 9 show that the coefficient of UHV is significantly positive in both groups, while the effect is significantly larger for enterprises with lower financing constraints. This suggests that improvements in energy infrastructure do not automatically translate into greater AI investment when financing constraints remain binding. Enterprises facing lower financing constraints are better able to convert improved electricity conditions into sustained AI investment and application. This points to a complementarity between infrastructure improvement and financial capacity: UHV transmission projects can alleviate energy-side constraints, but adequate financial resources remain important for realizing the innovation benefits of improved infrastructure.

**Table 9:** Misallocation correction effect results.

Variables	(1)	(2)	(3)	(4)
	High_SA	Low_SA	High_Lev	Low_Lev
	AI	AI	AI	AI
UHV	0.075**	0.122***	0.109*	0.062
	(0.037)	(0.046)	(0.056)	(0.043)
Controls	Yes	Yes	Yes	Yes
Year	Yes	Yes	Yes	Yes
Company	Yes	Yes	Yes	Yes
Constant	−3.162***	−2.964***	−3.646***	−2.512***
	(0.768)	(0.526)	(0.695)	(0.708)
N	19,782	20,377	19,571	20,226
Adj.R^2^	0.774	0.799	0.777	0.763
Difference in coefficient	0.047*		−0.047**	

Notes:***, **, and *, respectively, indicate that the coefficients are significant at the levels of 1%, 5%, and 10%. Standard errors in parentheses are clustered at the city level. Differences in coefficient are obtained by adopting the bootstrap-based Fisher permutation test with 1000 repeated sampling replications.

For the structural dimension, we use the asset-liability ratio (Lev) to capture capital structure pressure and divide enterprises into high- and low-leverage groups using the industry-year median. Columns (3)-(4) of Table 9 show that the coefficient of UHV is significantly positive for high-leverage enterprises, but insignificant for low-leverage enterprises, and the coefficient difference is statistically significant. This indicates that UHV transmission projects have greater value for enterprises facing higher debt pressure. Improved electricity reliability can reduce operating uncertainty and limit the crowding-out of long-term AI investment by short-term operating needs. In this sense, UHV transmission projects can partly mitigate the tendency to prioritize short-term operations over long-term innovation.

### 5.3. Shortboard complementing effect

The preceding analysis shows that UHV transmission projects can improve the allocation of energy and innovation resources. We further examine whether their benefits are greater where local development conditions provide weaker support for enterprises’ AI activities. Limitations in traditional electricity transmission may be particularly consequential in cities with weaker economic growth momentum or in less concentrated markets, where local economic and market conditions may provide relatively limited support for technology-intensive activities. By expanding the spatial reach of electricity supply and improving its stability, UHV transmission projects may partly compensate for these constraints and provide more favorable conditions for electricity- and computing-intensive AI activities. Therefore, we expect the marginal effect of UHV transmission projects to be greater where local development constraints are more binding.

To test this proposition, we consider two dimensions of local development conditions: urban economic growth (GDP) and market concentration (HHI). GDP is measured by the city-level GDP growth rate divided by 100, with a lower value indicating weaker local economic growth momentum. HHI is calculated as the sum of the squared shares of each enterprise’s owners’ equity in total industry owners’ equity, with a lower value indicating a less concentrated market structure. Based on Model (1), we construct Model (4) by introducing the interaction between UHV and the corresponding local development indicator:


$$\begin{array}{c} {AI}_{it}=\omega_{\text{0}}+\omega_{\text{1}}{UHV}_{it}+\omega_{\text{2}}T_{it}+\omega_{\text{3}}{UHV}_{it}\times T_{it}+\omega_{m}{Controls}_{it}+{Year}_{t}+{Company}_{i}+{\text{ℇ}}_{it} \end{array}$$ (4)

where, *T* represents proxy variables for development constraints at the city and market levels, including *GDP* and *HHI*. All other parameters are consistent with Model (1).

Table 10 reports the results. The coefficients of UHV×GDP and UHV×HHI are both significantly negative at the 5% level. This indicates that the positive effect of UHV transmission projects on enterprises’ AI development is greater in cities with weaker economic growth momentum and in less concentrated markets. The results suggest that the marginal value of energy infrastructure rises when local economic and market conditions provide relatively limited support for technological investment. By improving access to stable cross-regional electricity supply, UHV transmission projects can partly compensate for weaknesses in local development conditions and broaden the infrastructure base available for enterprises’ AI activities. In this sense, energy infrastructure not only improves resource allocation efficiency but may also help narrow differences in the external conditions supporting technological transformation.

**Table 10:** Shortboard complementing effect results.

Variables	(1)	(2)
	AI	AI
UHV	0.156***	0.131***
	(0.052)	(0.043)
GDP	0.597*	
	(0.353)	
UHV × GDP	−0.964**	
	(0.380)	
HHI		−0.256**
		(0.119)
UHV × HHI		−0.513**
		(0.251)
Controls	Yes	Yes
Year	Yes	Yes
Company	Yes	Yes
Constant	−3.033***	−2.954***
	(0.479)	(0.487)
N	40,450	40,445
Adj.R^2^	0.772	0.772

Notes:***, **, and *, respectively, indicate that the coefficients are significant at the levels of 1%, 5%, and 10%. Standard errors in parentheses are clustered at the city level.

## 6. Conclusions and implications

### 6.1. Conclusions

This study uses the staggered rollout of UHV transmission projects across Chinese prefecture-level cities as a quasi-natural experiment to examine how energy infrastructure affects enterprises’ AI development. The results show that UHV transmission projects significantly promote enterprises’ AI development, and this finding remains robust across alternative specifications, measurement approaches, and endogeneity tests. Further analysis based on the TOE framework indicates that energy infrastructure facilitates AI development through three complementary channels: strengthening enterprises’ technological innovation, expanding their organizational resource base, and improving regional financial development. The effects also vary across enterprises with different ownership, location, governance structures, and technological characteristics.

These findings reveal that the role of energy infrastructure extends beyond the conventional function of electricity supply. By improving the reliability and allocation of electricity resources, UHV transmission projects create favorable conditions for sustained technological investment and the adoption of computing-intensive technologies. More broadly, the evidence suggests that enterprises’ AI development depends not only on algorithms, data, and computing capabilities, but also on the physical infrastructure that supports their continuous operation. Therefore, energy infrastructure constitutes an important foundation for the diffusion and development of AI at the enterprise level.

### 6.2. Managerial and policy implications

Our findings have implications for both enterprise management and public policy. At the enterprise level, reliable electricity supply should be regarded not merely as a production input but as an important condition for sustained AI investment. Therefore, managers should incorporate energy security into long-term digital strategies and coordinate AI investment with R&D, computing capacity, and internal resource allocation. Such coordination can help enterprises better translate improvements in electricity infrastructure into technological innovation and organizational capacity, thereby strengthening the foundations for sustained AI development.

At the policy level, energy infrastructure planning should be more closely aligned with regional digital and innovation strategies. The heterogeneous effects identified in this study suggest that infrastructure investment should not follow a uniform approach. Cross-regional electricity allocation should be improved to alleviate structural mismatches, while greater attention should be directed toward regions facing weaker economic conditions or other development bottlenecks. For developing economies, the findings further indicate that AI policy should extend beyond algorithms, data, and computing facilities to include the energy systems that support their continuous operation. Where fiscal and resource constraints are binding, phased and regionally targeted infrastructure investment may provide a more feasible path toward building the physical foundations for enterprises’ AI development.

### 6.3. Limitations and research prospects

Despite these findings, several limitations remain. First, the sample is restricted to Chinese listed enterprises, which generally have greater financing capacity, organizational resources, and technological capabilities than smaller or non-listed firms. Therefore, the findings may not fully capture how energy infrastructure affects AI development among enterprises facing more severe resource constraints. Future research could incorporate non-listed enterprises or survey-based microdata to examine whether the effects vary across a broader range of enterprises. Second, although we conduct several measurement-based robustness tests, the annual-report indicator remains a disclosure-based proxy for AI development. Future studies could combine textual measures with AI-related patents, investment, recruitment, or actual technology deployment data to provide a more multidimensional assessment. Finally, the longer-term and broader spillover effects of UHV transmission projects remain insufficiently explored. Energy infrastructure may influence AI development beyond directly connected enterprises through supply chains, industrial linkages, and cross-regional knowledge or resource flows. Extending the observation period and incorporating spatial or network-based approaches would help clarify the persistence and wider diffusion of these effects.

## Supporting information

S1 File Appendix. (DOCX)
