# Supplementary material for: Energy infrastructure construction and enterprises’ Artificial Intelligence development: Evidence from China’s ultra-high voltage transmission projects
Source: PLoS One. 2026 Oct 1;21(10):e0355269. doi: 10.1371/journal.pone.0355269 (PMC13630311; doi:10.1371/journal.pone.0355269)
Supplement: Supplementary file 1 [file pone.0355269.s001.docx]

Appendix

Table 1 Opening time and generation type of UHV lines from 2009 to 2025.

| Time | Line Name | Line Length  (km) | Investment  (100 million RMB) | Covered Prefecture-level Cities |
| --- | --- | --- | --- | --- |
| 2009.01 | Jindongnan-Nanyang-Jingmen 1000 kV UHVAC Project | 640 | 58 | Changzhi (Shanxi), Nanyang (Henan), Jingmen (Hubei) |
| 2010.06 | Yunnan-Guangzhou ±800 kV UHVDC Project | 1373 | 137 | Chuxiong (Yunnan), Guangzhou (Guangdong) |
| 2010.07 | Xiangjiaba-Shanghai ±800 kV UHVDC Project | 1908 | 233 | Yibin (Sichuan), Shanghai |
| 2012.12 | Jinping-Sunan ±800 kV UHVDC Project | 2059 | 220 | Liangshan (Sichuan), Suzhou (Jiangsu) |
| 2013.09 | Huainan-Zhebei-Shanghai 1000 kV UHVAC Project | 1312 | 192 | Huainan, Wuhu (Anhui), Huzhou (Zhejiang), Shanghai |
| 2014.01 | Hainan-Zhengzhou ±800 kV UHVDC Project | 2210 | 234 | Hami (Xinjiang), Zhengzhou (Henan) |
| 2014.07 | Xiluodu Left Bank-Zhejiang ±800 kV UHVDC Project | 1562 | 197 | Yibin (Sichuan), Jinhua (Zhejiang) |
| 2014.12 | Zhebei-Fuzhou 1000 kV UHVAC Project | 1206 | 197 | Lishui, Jinhua (Zhejiang), Fuzhou (Fujian) |
| 2015.05 | Nuozhadu-Guangdong ±800 kV UHVDC Project | 1413 | 160 | Pu’er (Yunnan), Jiangmen (Guangdong) |
| 2016.07 | Ximeng-Shandong 1000 kV UHVAC Project | 1460 | 177 | Xilingol League (Inner Mongolia), Chengde (Hebei), Beijing, Jinan (Shandong) |
| 2016.09 | Ningxia Lingzhou-Zhengjiang Shaoxing ±800 kV UHVDC Project | 1720 | 219 | Yinchuan (Ningxia), Shaoxing (Zhejiang) |
| 2016.11 | Mengxi-Tianjin South 1000 kV UHVAC Project | 1216 | 173 | Ordos (Inner Mongolia), Jinzhong (Shanxi), Tianjin |
| 2016.11 | Huainan-Nanjing-Shanghai 1000 kV UHVAC Project | 1560 | 272 | Huainan, Bengbu (Anhui), Nanjing, Suzhou, Taizhou (Jiangsu), Shanghai |
| 2017.06 | Jiuquan-Hunan ±800 kV UHVDC Project | 2386 | 259 | Jiuquan (Gansu), Xiangtan (Hunan) |
| 2017.06 | Jinbei-Jiangsu ±800 kV UHVDC Project | 1111 | 158 | Shuozhou (Shanxi), Huai’an (Jiangsu) |
| 2017.08 | Yuheng-Weifang 1000 kV UHVAC Project | 2098 | 227 | Yulin (Shaanxi), Jinzhong (Shanxi), Shijiazhuang (Hebei), Jinan, Weifang (Shandong) |
| 2017.08 | Ximeng-Shengli 1000 kV UHVAC Project | 480 | 50 | Xilingol League (Inner Mongolia), Zhangjiakou (Hebei) |
| 2017.10 | Ximeng-Taizhou ±800 kV UHVDC Project | 1628 | 232 | Xilingol League (Inner Mongolia), Taizhou (Jiangsu) |
| 2017.12 | Zhalute-Qingzhou ±800 kV UHVDC Project | 1234 | 221 | Tongliao (Inner Mongolia), Weifang (Shandong) |
| 2018.05 | Northwest Yunnan-Guangdong ±800 kV UHVDC Project | 1959 | 222 | Dali (Yunnan), Shenzhen (Guangdong) |
| 2019.01 | Shanghai Temple-Shandong ±800 kV UHVDC Project | 1230 | 221 | Ordos (Inner Mongolia), Linyi (Shandong) |
| 2019.06 | Beijing West-Shijiazhuang 1000 kV UHVAC Project | 456 | 34 | Beijing, Shijiazhuang (Hebei) |
| 2019.09 | Sutong 1000 kV GIL Integrated Pipeline 1000 kV UHVAC Project | 35 | 48 | Suzhou, Nantong (Jiangsu) |
| 2019.09 | Zhundong-Wannan ±1100 kV UHVDC Project | 3324 | 407 | Changji (Xinjiang), Xuancheng (Anhui) |
| 2020.01 | Shangdong-Hebei Loop Network 1000 kV UHVAC Project | 1639 | 152 | Heze, Linyi, Zaozhuang (Shandong), Xingtai (Hebei) |
| 2020.08 | Zhangbei-Xiongan 1000 kV UHVAC Project | 639.8 | 59.8 | Zhangjiakou, Baoding (Hebei) |
| 2020.09 | Mengxi-Jinzhong 1000 kV UHVAC Project | 608 | 49 | Ordos (Inner Mongolia), Jinzhong (Shanxi) |
| 2020.12 | Qinghai-Henan ±800 kV UHVDC Project | 1587 | 271 | Hainan (Qinghai), Zhumadian (Henan) |
| 2020.12 | Wudongde-Guangdong-Guangxi ±800 kV UHVDC Project | 1452 | 242.49 | Kunming (Yunnan), Liuzhou (Guangxi), Huizhou (Guangdong) |
| 2020.12 | Zhumadian-Nanyang 1000 kV UHVAC Project | 188.4 | 21.6 | Zhumadian, Nanyang (Henan) |
| 2021.06 | Yazhong-Jiangxi ±800 kV UHVDC Project | 1711 | 244 | Liangshan (Sichuan), Fuzhou (Jiangxi) |
| 2021.12 | Nanchang-Changsha 1000 kV UHVAC Project | 690.4 | 104 | Nanchang (Jiangxi), Changsha (Hunan) |
| 2022.04 | Northern Shaanxi-Wuhan ±800 kV UHVDC Project | 1137 | 185 | Yulin (Shaanxi), Wuhan (Hubei) |
| 2022.07 | Baihetan-Jiangsu ±800 kV UHVDC Project | 2087 | 307 | Liangshan (Sichuan), Suzhou (Jiangsu) |
| 2022.10 | Nanyang-Jingmen-Changsha 1000 kV UHVAC Project | 624.8 | 83.6 | Nanyang (Henan), Jingmen (Hubei), Changsha (Hunan) |
| 2022.12 | Jingmen-Wuhan 1000 kV UHVAC Project | 476 | 65.4 | Jingmen, Wuhan (Hubei) |
| 2023.06 | Baihetan-Zhejiang ±800 kV UHVDC Project | 2120 | 299 | Liangshan (Sichuan), Hangzhou (Zhejiang) |
| 2023.11 | Zhumadian-Wuhan 1000 kV UHVAC Project | 562 | 38 | Zhumadian (Henan), Wuhan (Hubei) |
| 2023.12 | Fuzhou-Xiamen 1000 kV UHVAC Project | 476 | 71.2 | Fuzhou, Xiamen (Fujian) |
| 2024.10 | Zhangbei-Shengli 1000 kV UHVAC Project | 732 | 67.86 | Zhangjiakou (Hebei), Xilinhot (Inner Mongolia) |
| 2024.11 | Wuhan-Nanchang 1000 kV UHVAC Project | 925.8 | 86.6 | Wuhan (Hubei), Nanchang (Jiangxi) |
| 2024.11 | Longdong-Shandong ±800 kV UHVDC Project | 926 | 202 | Qingyang (Gansu), Tai’an (Shandong) |
| 2024.12 | Sichuan-Chongqing 1000 kV UHVAC Project | 1316 | 286.31 | Chengdu, Ya’an (Sichuan), Chongqing |
| 2025.06 | Hami-Chongqing ±800 kV UHVDC Project | 2290 | 286 | Hami (Xinjiang), Chongqing |
| 2025.08 | Ningxia-Hunan ±800 kV UHVDC Project | 1616 | 281 | Zhongwei (Ningxia), Hengyang (Hunan) |
| 2025.12 | Jinshang-Hubei ±800 kV UHVDC Project | 1901 | 334.8 | Qamdo (Xizang), Huangshi (Hubei) |
